# Supplementary material for: MAGIs regulate aPKC to enable balanced distribution of intercellular tension for epithelial sheet homeostasis
Source: Commun Biol. 2021 Mar 12;4:337. doi: 10.1038/s42003-021-01874-z (PMC7954791; doi:10.1038/s42003-021-01874-z)
Supplement: Supplementary file 2 — Supplementary Information [file 42003_2021_1874_MOESM2_ESM.pdf]

# MAGIs regulate aPKC to enable balanced distribution of  intercellular tension for epithelial sheet homeostastis

Kenji Matsuzawa, Hayato Ohga, Kenta Shigetomi, Tomohiro Shiiya, Masanori Hirashima, Junichi Ikenouchi

**Supplementary Information**

- Supplementary Figures S1-S8
- Supplementary Data 1

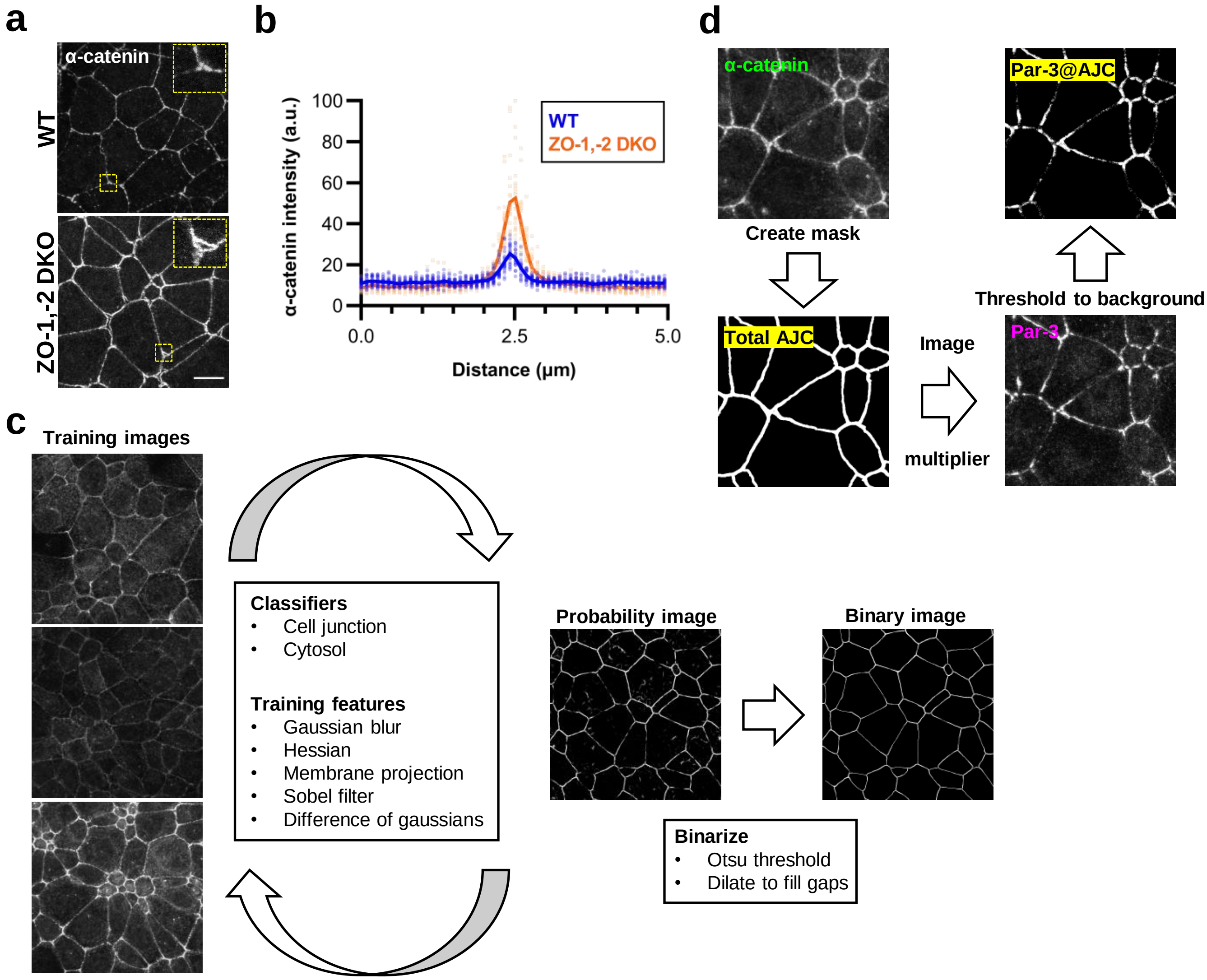


**Supplementary Figure S1 (Related to Fig. 1)**
**Details of the image analysis methodologies**

**a** Representative immunofluorescence images of WT and ZO-1,-2 DKO cells stained for activated α-catenin. Inset are magnified images of tricellular contacts. Scale bar, 10 μm.
**b** Cross-junctional line scans of activated α-catenin from images corresponding to **a**. Individual data from 20 independent line scans are shown with the means depicted by solid lines. Source data are available in **Supplementary Data 1**.
**c** Schematic of the image segmentation protocol. Representative images were processed to reduce noise and enhance edges by applying the indicated filters. Image pixels were then manually classified as either 'Cell junction' or 'Cytosol' to train and refine the protocol until automated segmentation images for new input images adequately delineated cell junctions. The probability images thus obtained were binarized and dilated to eliminate discontinuous cell junctions.
**d** Workflow for analyzing immunofluorescence specifically at AJC. The segmented image (total AJC) was multiplied over the immunofluorescence image of interest and a threshold was applied to the resulting image to remove non-AJC background. The remaining signals were measured to obtain mean intensity and coverage area data.

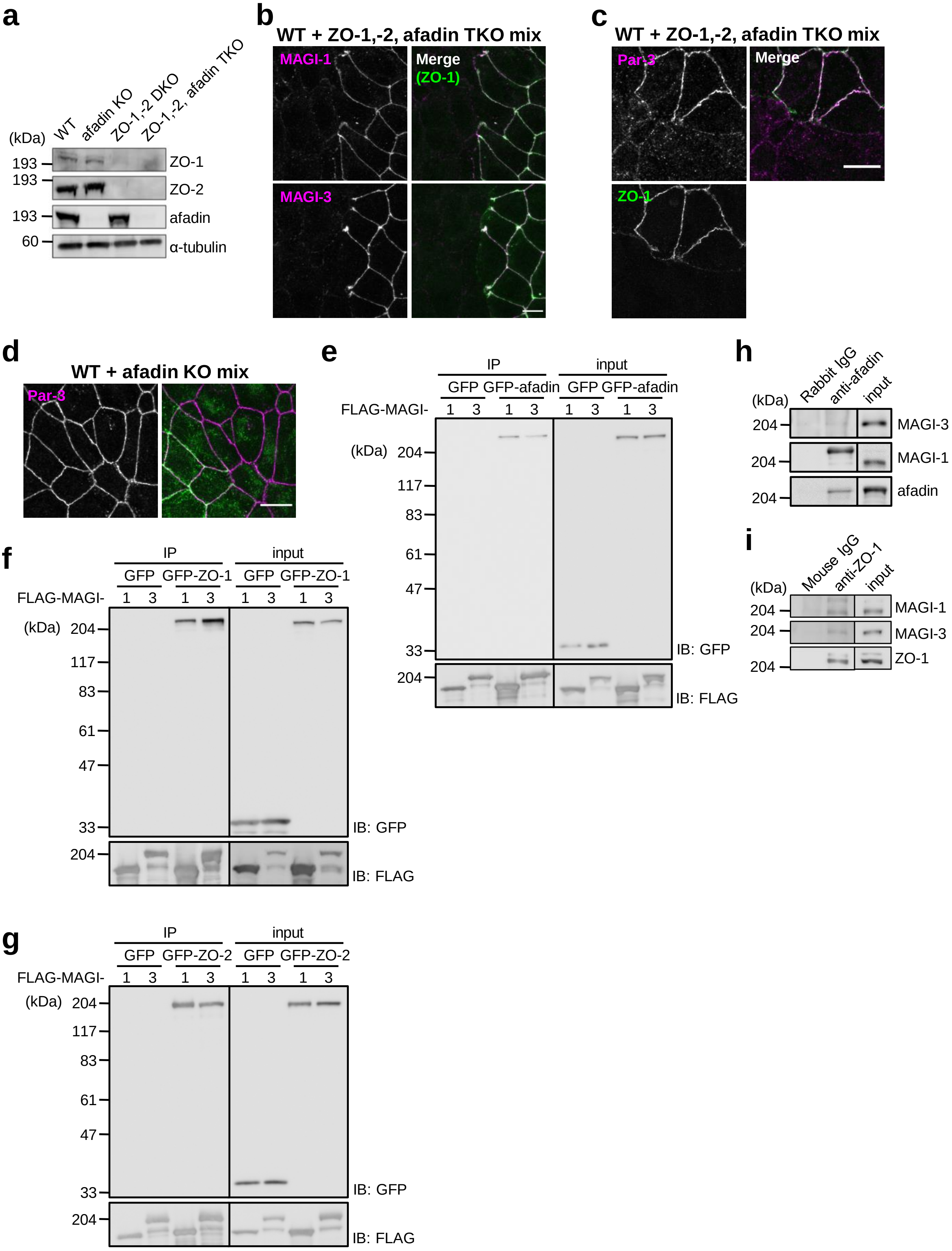
a
(kDa)
WT
afadin KO
ZO-1,-2 DKO
ZO-1,-2, afadin TKO
193
193
193
60
ZO-1
ZO-2
afadin
α-tubulin
b
WT + ZO-1,-2, afadin TKO mix
MAGI-1
Merge
(ZO-1)
MAGI-3
c
WT + ZO-1,-2, afadin TKO mix
Par-3
Merge
ZO-1
d
WT + afadin KO mix
Par-3
e
IP
input
GFP
GFP-afadin
GFP
GFP-afadin
FLAG-MAGI-
1 3 1 3 1 3 1 3
(kDa)
204
117
83
61
47
33
204
IB: GFP
IB: FLAG
f
IP
input
GFP
GFP-ZO-1
GFP
GFP-ZO-1
FLAG-MAGI-
1 3 1 3 1 3 1 3
(kDa)
204
117
83
61
47
33
204
IB: GFP
IB: FLAG
g
IP
input
GFP
GFP-ZO-2
GFP
GFP-ZO-2
FLAG-MAGI-
1 3 1 3 1 3 1 3
(kDa)
204
117
83
61
47
33
204
IB: GFP
IB: FLAG
h
(kDa)
Rabbit IgG
anti-afadin
input
204
204
204
MAGI-3
MAGI-1
afadin
i
(kDa)
Mouse IgG
anti-ZO-1
input
204
204
204
MAGI-1
MAGI-3
ZO-1

**Supplementary Figure S2 (Related to Fig. 2)**
**ZO proteins and afadin differentially recruit MAGI to AJC**

**a** Lysates of EpH4 WT, ZO-1,-2 DKO and ZO-1,-2, afadin TKO cells were immunoblotted with the indicated antibodies to confirm protein depletion. Alpha-tubulin is the loading control.

**b** Representative immunofluorescence images of a co-culture of WT and ZO-1,-2, afadin TKO cells stained for either MAGI-1 (magenta, upper panel) or MAGI-3 (magenta, lower panel) with ZO-1 (green). Scale bar, 10 μm.

**c** Representative immunofluorescence images of a co-culture of WT and ZO-1,-2, afadin TKO cells stained for Par-3 (magental with ZO-1 (green). Scale bar, 10 μm.

**d** Representative immunofluorescence images of a co-culture of WT and afadin KO cells stained for Par-3 (magenta) and afadin (green). Scale bar, 10 μm.

**e** FLAG-MAGI and GFP-afadin were heterologously expressed in HEK293 cells. Inputs and FLAG immunoprecipitates were immunoblotted with the indicated antibodies.

**f** FLAG-MAGI and GFP-ZO-1 were heterologously expressed in HEK293 cells. Inputs and FLAG immunoprecipitates were immunoblotted with the indicated antibodies.

**g** FLAG-MAGI and GFP-ZO-2 were heterologously expressed in HEK293 cells. Inputs and FLAG immunoprecipitates were immunoblotted with the indicated antibodies.

**h** and **i** Endogenous afadin (**h**) or ZO-1 (**i**) was immunoprecipitated from EpH4 WT cell lysates. Input and immunoprecipitates were immunoblotted with the indicated antibodies. Uncropped immunoblots are shown in **Supplementary Figure S7**.

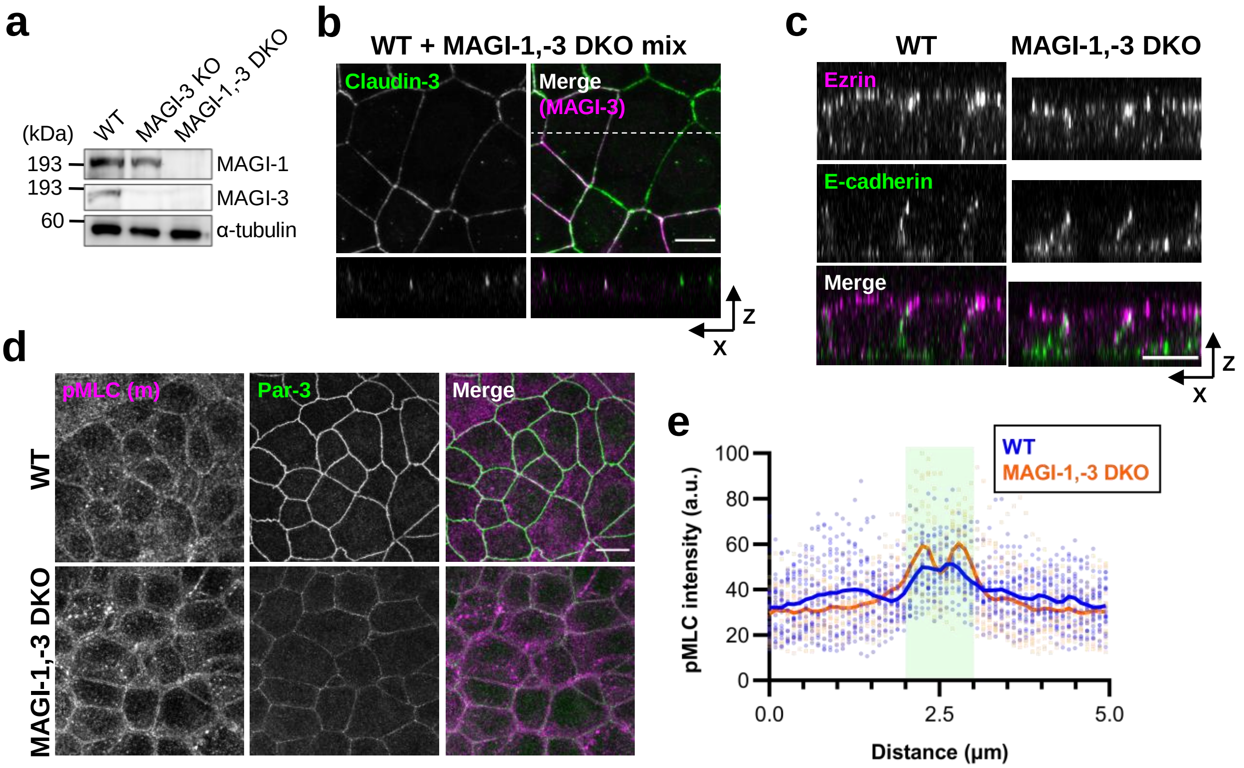


**Supplementary Figure S3 (related to Fig. 2)**
**Epithelial polarization is unaltered in MAGI-1,-3 DKO cells**

**a** Lysates of EpH4 WT, MAGI-3 KO and MAGI-1,-3 DKO cells were immunoblotted with the indicated antibodies to confirm protein depletion. Alpha-tubulin is the loading control. Uncropped immunoblots are shown in **Supplementary Figure S8**.
**b** Representative immunofluorescence images of a co-culture of WT and MAGI-1,-3 DKO cells stained for Claudin-3 (green) and MAGI-3 (magenta). Scale bar, 10 μm.
**c** Representative immunofluorescence images of WT and MAGI-1,-3 DKO cells stained for the apical marker Ezrin (magenta) and the basolateral marker E-cadherin (green). Scale bar, 10 μm.
**d** Representative immunofluorescence images of WT and MAGI-1,-3 DKO cells stained for pMLC (magenta) and Par-3 (green). Scale bar, 10 μm.
**e** Cross-junctional line scans of pMLC immunofluorescence in images corresponding to **d**. Shaded area represents AJC as defined by the Par-3 peak. Individual data from 20 independent line scans are shown with the means depicted by solid lines. Source data are available in **Supplementary Data 1**.

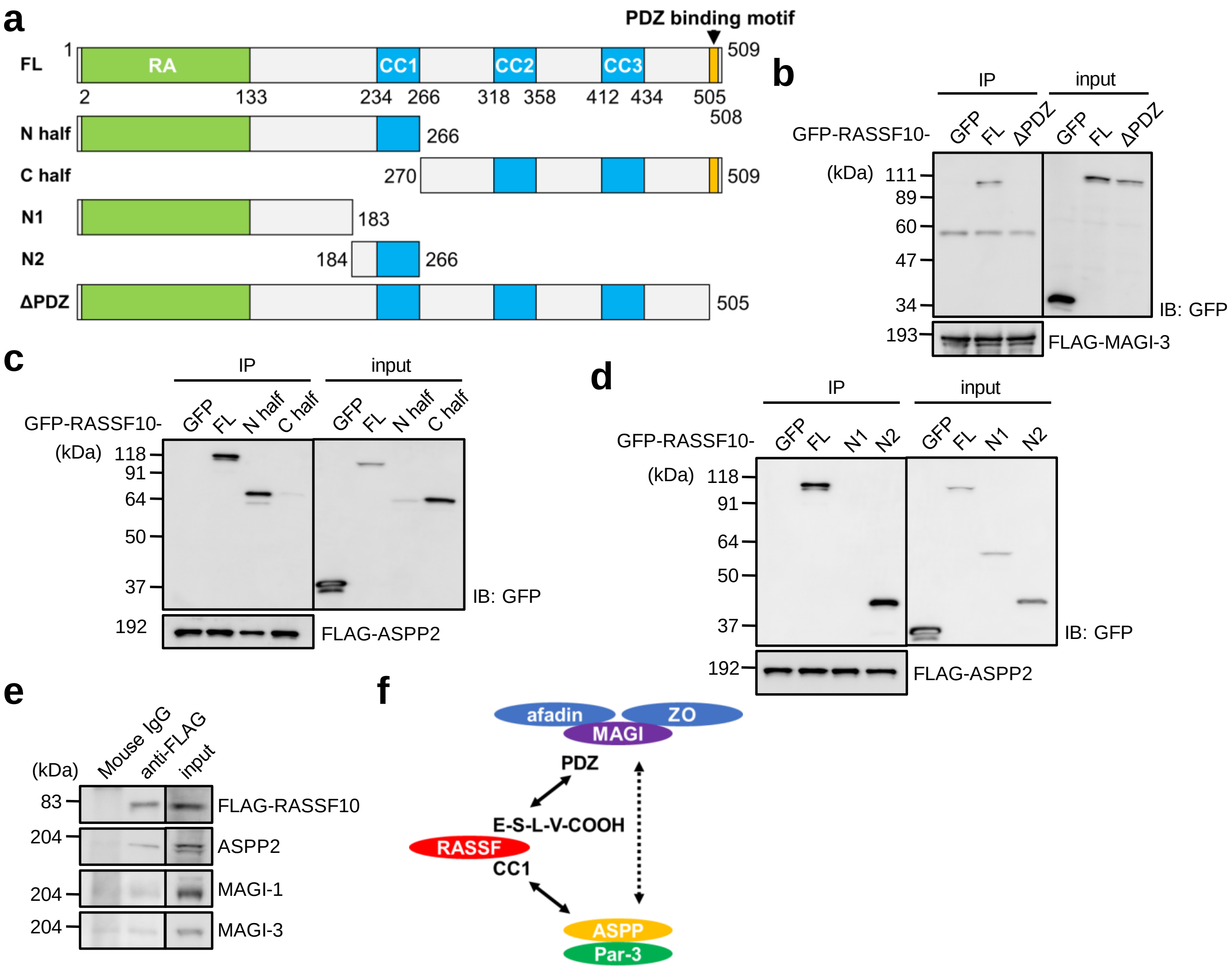


**Supplementary Figure S4 (related to Fig. 3)**

**MAGI-3 forms a ternary complex with the Par-3 regulator ASPP2 and the N-terminal RASSF protein RASSF10**

**a** Domain structure of RASSF10 and schematic of the deletion mutants used for binding studies. The numbers below indicate amino acid residues. RA; RA domain, CC 1-3; Coiled-coil domains 1-3. The potential PDZ binding motif is indicated at amino acid residues 505 through 508.

**b** FLAG-MAGI-3 and GFP-RASSF10, either WT or a deletion lacking the potential PDZ binding motif (ΔPDZ), were heterologously expressed in HEK293 cells. Input and FLAG immunoprecipitates were probed with the indicated antibodies.

**c** and **d** FLAG-ASPP2 and the indicated GFP-RASSF10 fragments were heterologously expressed in HEK293 cells. Input and FLAG immunoprecipitates were probed with the indicated antibodies.

**e** FLAG immunoprecipitation from lysates of EpH4 cells stably expressing FLAG-RASSF10. Input and immunoprecipitates were immunoblotted with the indicated antibodies. Uncropped immunoblots are shown in **Supplementary Figure S8**.

**f** Model showing MAGI in complex with ASPP-Par-3. MAGI can directly interact with ASPP or form a ternary complex through RASSF10.

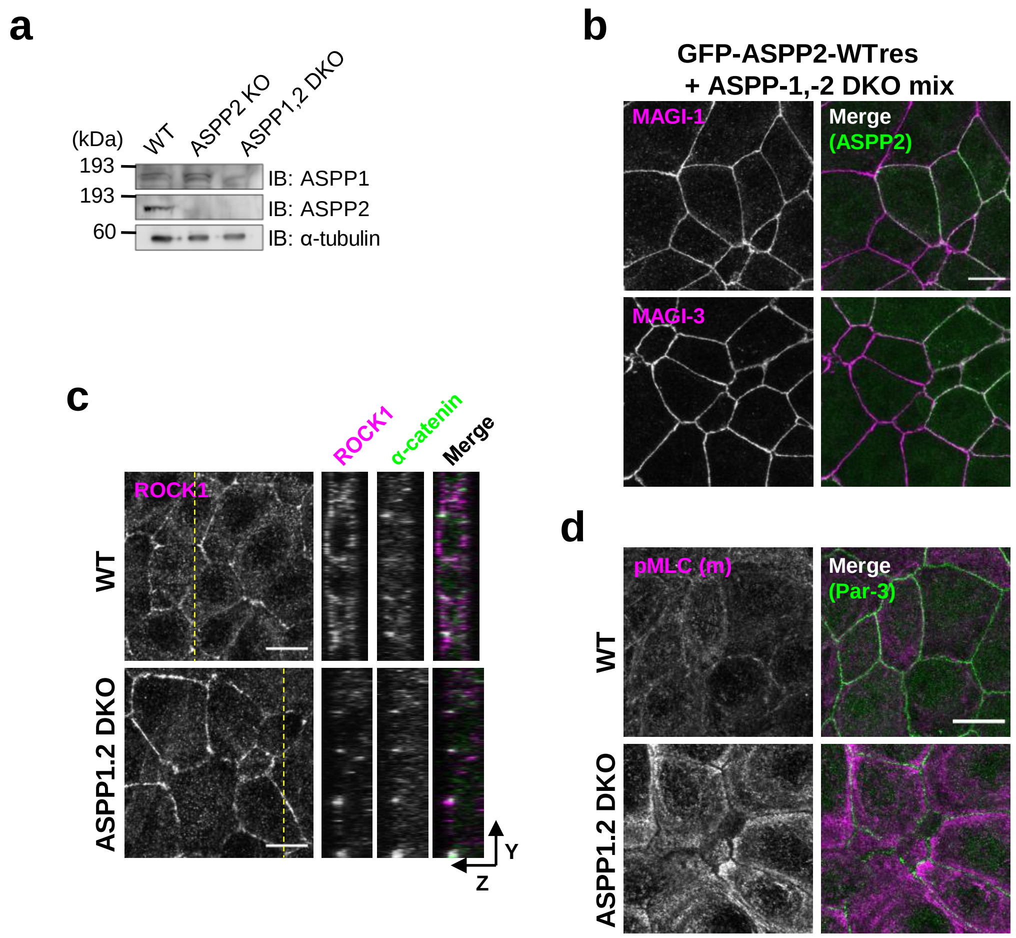


**Supplementary Figure S5 (related to Fig. 3)**

**MAGI act through ASPP2 to control ROCK1-dependent apical contractility**

**a** Lysates of EpH4 WT, ASPP2 KO and ASPP1,2 DKO cells were immunoblotted with the indicated antibodies to confirm protein depletion. Alpha-tubulin is the loading control. Uncropped immunoblots are shown in **Supplementary Figure S8**.

**b** Representative immunofluorescence images of a co-culture of ASPP1,2 DKO and GFP-ASPP2-WTres cells stained for either MAGI-1 (magenta, upper panel) or MAGI-3 (magenta, lower panel) with GFP (ASPP2; green). Scale bar, 10 μm.

**c** Representative immunofluorescence images of WT and ASPP1,2 DKO cells stained for ROCK1 (magenta) and activated α-catenin (green). Scale bar, 10 μm.

**d** Representative immunofluorescence images of WT and ASPP1,2 DKO cells stained for pMLC (magenta) and Par-3 (green). Scale bar, 10 μm.

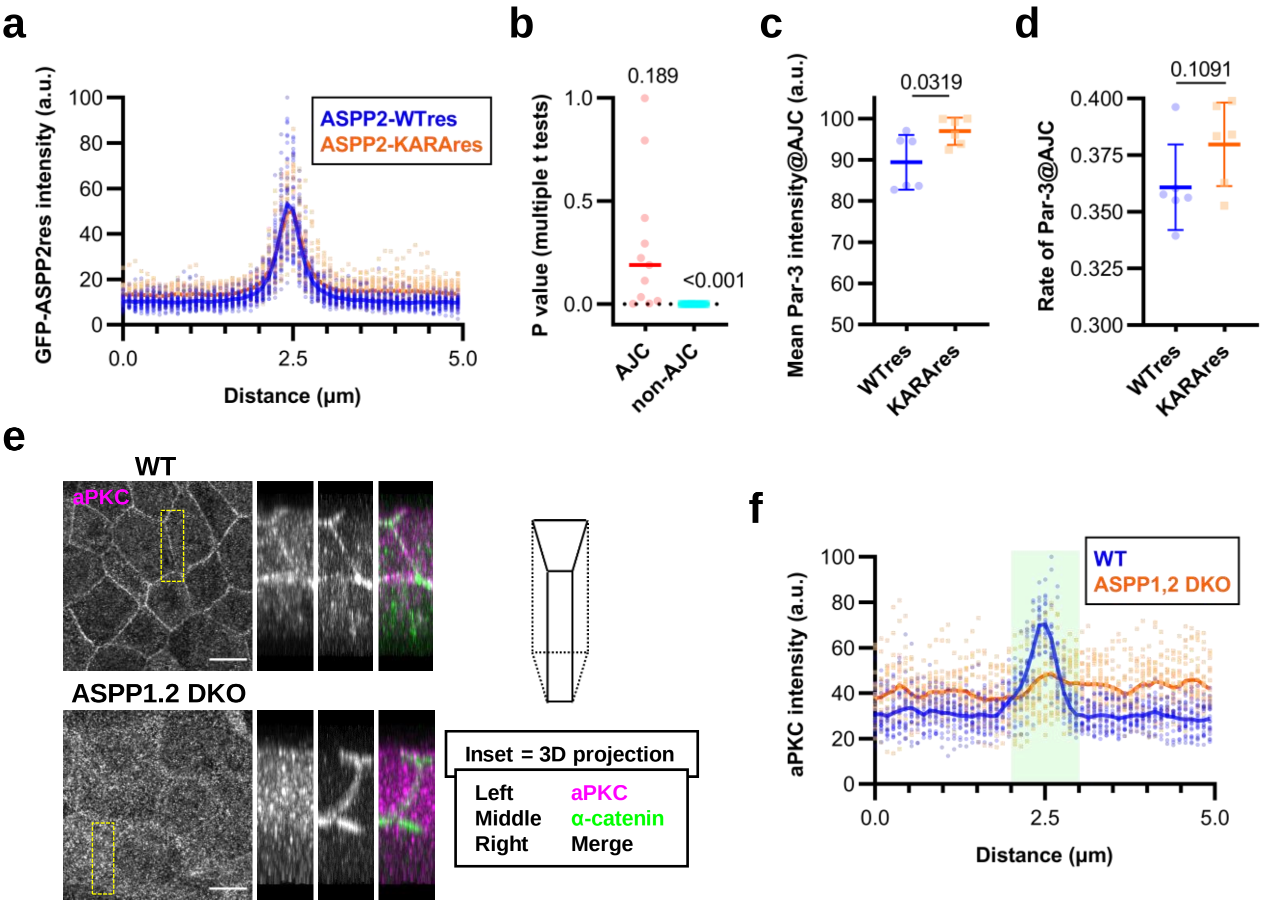


**Supplementary Figure S6 (related to Fig. 4)**
**Par-3 localization to AJC is restomagenta in ASPP2 rescue cells independent of PP1 interaction**

**a** Cross-junctional line scans of GFP fluorescence in images corresponding to **Fig. 4a**, **f** and **h**. Individual data from 60 independent line scans are shown with the means depicted by solid lines.

**b** P values of multiple t tests comparing GFP fluorescence in GFP-ASPP2-WTres and GFP-ASPP2-KARAres cells. AJC values were those obtained between 2 and 3 μm; all other values, presumably corresponding to the lateral membrane, were considered non-AJC. Medians are notated and shown graphically as lines.

**c** Quantification of Par-3 mean fluorescence intensities at AJC in GFP-ASPP2-WTres and GFP-ASPP2-KARAres cells from images represented in **Fig. 4a**. P value from unpaired t test is shown.

**d** Quantification of Par-3 coverage relative to total AJC area based on data obtained with **c**. P value from unpaired t test is shown.

**e** Representative immunofluorescence images of WT and ASPP1,2 DKO cells stained for aPKC (magenta) and activated α-catenin (green). 3D projections of the region indicated by the yellow dotted square are shown to the right. Scale bar, 10 μm.

**f** Cross-junctional line scans of aPKC immunofluorescence from images corresponding to **e**. Shaded area represents AJC as defined by the activated α-catenin peak. Individual data from 20 independent line scans are shown with the means depicted by solid lines.

Source data are available in **Supplementary Data 1**.

## Fig. 3c

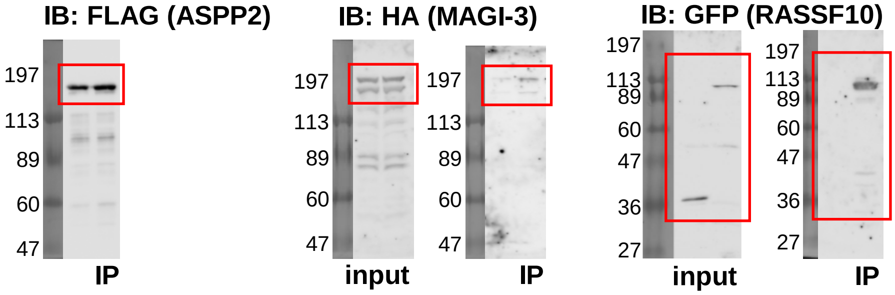


## Supplementary Figure S2a

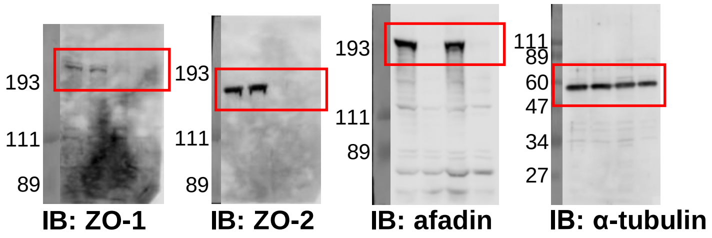


## Supplementary Figure S2h

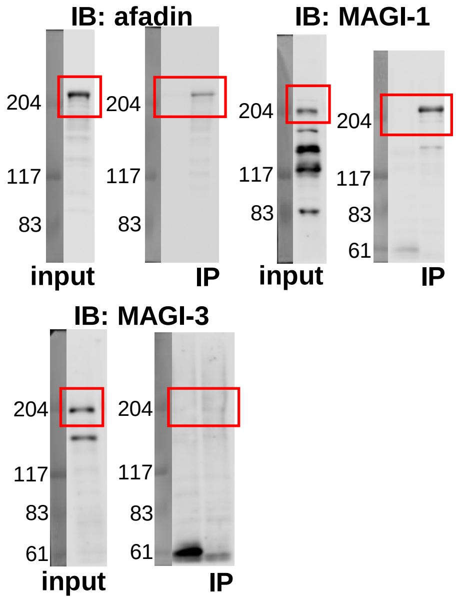


## Supplementary Figure S2e

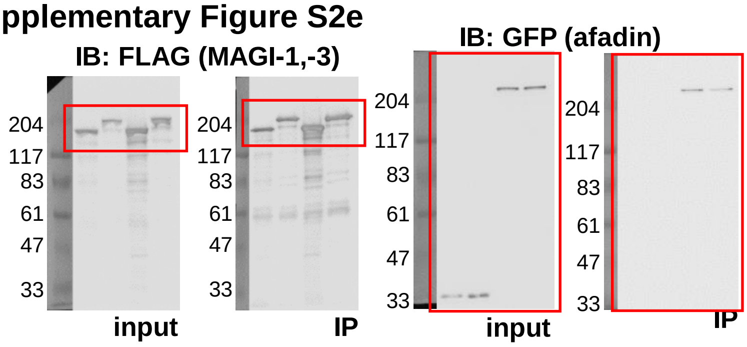


## Supplementary Figure S2f

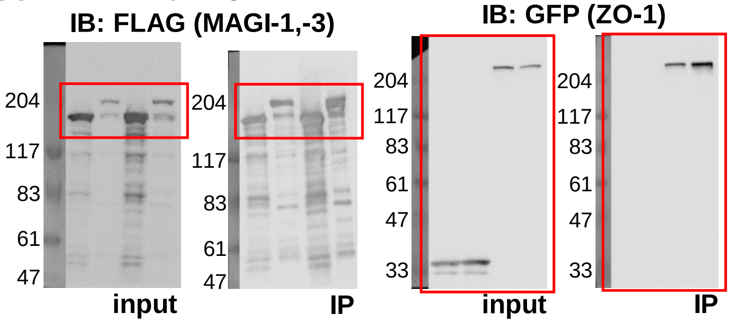


## Supplementary Figure S2i

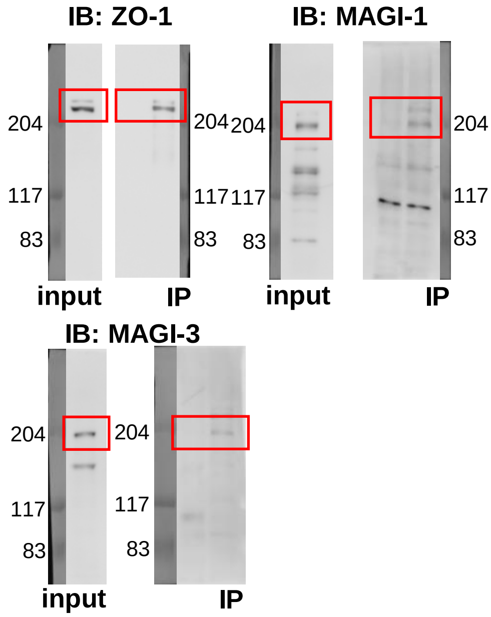


## Supplementary Figure S2g

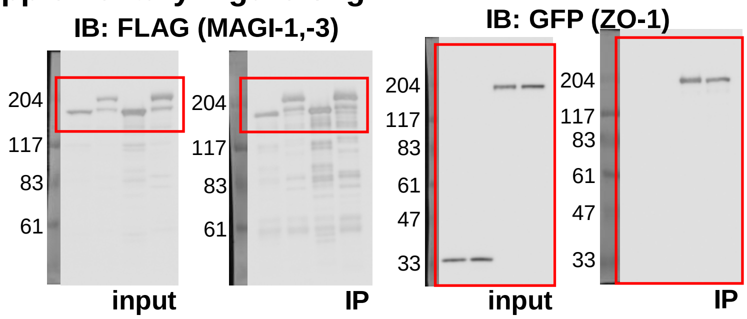


**Supplementary Figure S7 (related to Fig. 3 and Supplementary Figure S2)**
**Uncropped immunoblots**

## Supplementary Figure S3a

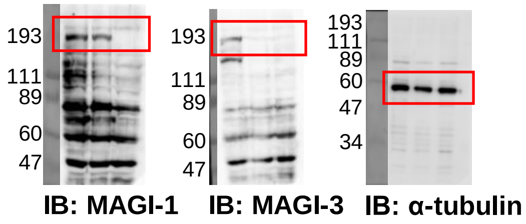


## Supplementary Figure S5a

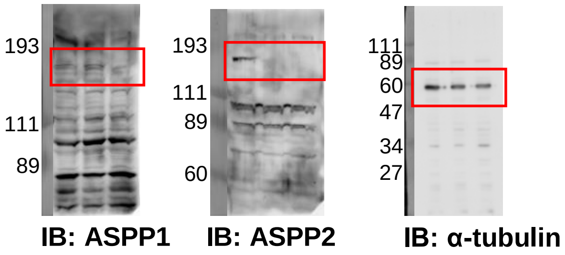


## Supplementary Figure S4b

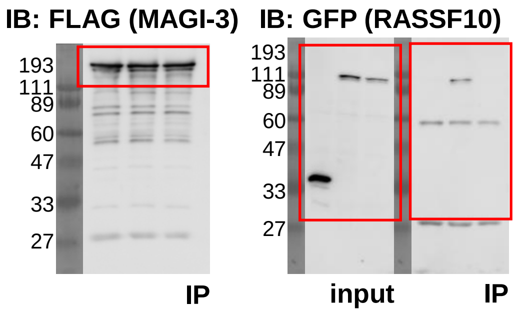


## Supplementary Figure S4c

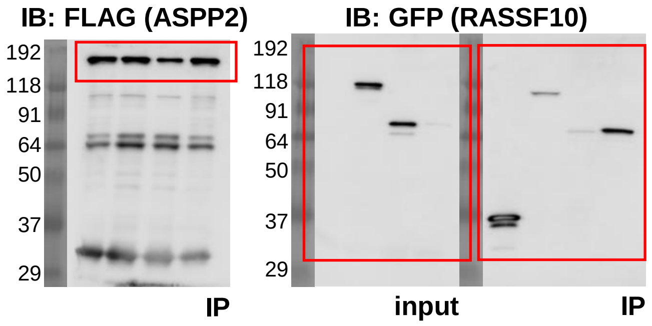


## Supplementary Figure S4d

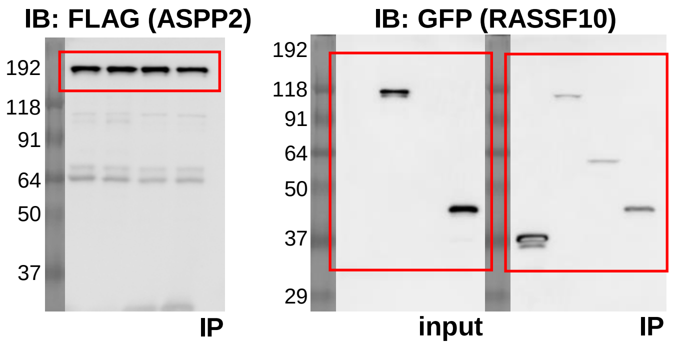


## Supplementary Figure S4e

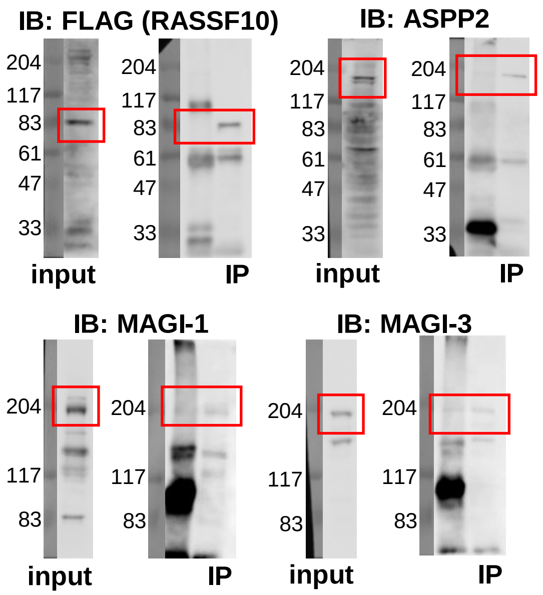


**Supplementary Figure S8 (related to Supplementary Figures S3, S4, S5)**
**Uncropped immunoblots**
