# Supplementary material for: MAGIs regulate aPKC to enable balanced distribution of intercellular tension for epithelial sheet homeostasis
Source: Commun Biol. 2021 Mar 12;4:337. doi: 10.1038/s42003-021-01874-z (PMC7954791; doi:10.1038/s42003-021-01874-z)
Supplement: Supplementary file 3 — Description of Additional Supplementary Files [file 42003_2021_1874_MOESM3_ESM.pdf]

Description of Additional Supplementary Files

File name: Supplementary Data 1

Description: The binarized cell images related to Fig. 1d-f, Fig. 2b-e, Fig. 3e-h and Fig. 4b-e and the source data for the graphs presented in Fig. 1c, e, g, f, i, k, l; Fig. 2c, d, e, g, i, j, k; Fig. 3b, d, f, g, h, j, k, l; Fig. 4c, d, e, g, i; Fig. 5a, b; Supplementary Figure S3e; Supplementary Figure S6a, b, c, d, f.
