# Supplementary material for: MAGIs regulate aPKC to enable balanced distribution of intercellular tension for epithelial sheet homeostasis
Source: Commun Biol. 2021 Mar 12;4:337. doi: 10.1038/s42003-021-01874-z (PMC7954791; doi:10.1038/s42003-021-01874-z)
Supplement: Supplementary file 5 — Reporting Summary [file 42003_2021_1874_MOESM5_ESM.pdf]

nature research

Corresponding author(s): Junichi Ikenouchi

Last updated by author(s): Feb 3, 2021

Reporting Summary

Nature Research wishes to improve the reproducibility of the work that we publish. This form provides structure for consistency and transparency in reporting. For further information on Nature Research policies, see our Editorial Policies and the Editorial Policy Checklist.

Statistics

For all statistical analyses, confirm that the following items are present in the figure legend, table legend, main text, or Methods section.

n/a	Confirmed	
☐	☒	The exact sample size (*n*) for each experimental group/condition, given as a discrete number and unit of measurement
☐	☒	A statement on whether measurements were taken from distinct samples or whether the same sample was measured repeatedly
☐	☒	The statistical test(s) used AND whether they are one- or two-sided<br>*Only common tests should be described solely by name; describe more complex techniques in the Methods section.*
☒	☐	A description of all covariates tested
☐	☒	A description of any assumptions or corrections, such as tests of normality and adjustment for multiple comparisons
☐	☒	A full description of the statistical parameters including central tendency (e.g. means) or other basic estimates (e.g. regression coefficient) AND variation (e.g. standard deviation) or associated estimates of uncertainty (e.g. confidence intervals)
☐	☒	For null hypothesis testing, the test statistic (e.g. *F*, *t*, *r*) with confidence intervals, effect sizes, degrees of freedom and *P* value noted<br>*Give P values as exact values whenever suitable.*
☒	☐	For Bayesian analysis, information on the choice of priors and Markov chain Monte Carlo settings
☒	☐	For hierarchical and complex designs, identification of the appropriate level for tests and full reporting of outcomes
☒	☐	Estimates of effect sizes (e.g. Cohen's *d*, Pearson's *r*), indicating how they were calculated

Our web collection on statistics for biologists contains articles on many of the points above.

Software and code

Policy information about availability of computer code

Data collection	ZEN2012 (Zeiss) was used to collect data from confocal microscopy.
Data analysis	Cell image analysis was performed with ImageJ.<br>GraphPad Prism 8.4.1 (GraphPad Software) was used to graph data and to perform statistical analyses.

For manuscripts utilizing custom algorithms or software that are central to the research but not yet described in published literature, software must be made available to editors and reviewers. We strongly encourage code deposition in a community repository (e.g. GitHub). See the Nature Research guidelines for submitting code & software for further information.

Data

Policy information about availability of data

All manuscripts must include a data availability statement. This statement should provide the following information, where applicable:

- Accession codes, unique identifiers, or web links for publicly available datasets
- A list of figures that have associated raw data
- A description of any restrictions on data availability

The datasets generated during and/or analysed during the current study are available from the corresponding author on reasonable request.

Field-specific reporting

Life sciences study design

All studies must disclose on these points even when the disclosure is negative.

Sample size	The sample size was chosen in advance on the basis of common practice of the described experiment and is mentioned for each experiment. No statistical methods were used to predetermine sample size. We ensured that sample sizes were sufficient by checking that inclusion of additional data points did not significantly change the variance (SD) of the data.
Data exclusions	No data were excluded.
Replication	All experimental results were replicated at least 3 times with enough reproducibility. Therefore, attempts of data replication were successful.
Randomization	Animals and humans were not used in the study. Randomization is not relevant as the same cell lines were used in all cases.
Blinding	Blinding is not applicable to data collection. In the case of time-lapse imaging analysis, all labels were removed and individual microscopy files were analyzed blindly in ImageJ.

Reporting for specific materials, systems and methods

We require information from authors about some types of materials, experimental systems and methods used in many studies. Here, indicate whether each material, system or method listed is relevant to your study. If you are not sure if a list item applies to your research, read the appropriate section before selecting a response.

Materials & experimental systems

n/a	Involved in the study	
☐	☒	Antibodies
☐	☒	Eukaryotic cell lines
☒	☐	Palaeontology and archaeology
☒	☐	Animals and other organisms
☒	☐	Human research participants
☒	☐	Clinical data
☒	☐	Dual use research of concern

Methods

n/a	Involved in the study	
☒	☐	ChIP-seq
☒	☐	Flow cytometry
☒	☐	MRI-based neuroimaging

Antibodies

Antibodies used	The following primary antibodies were used for immunofluorescence microscopy, immunoprecipitation and immunoblotting: rat anti-ZO-1 (DSHB); rabbit anti-ZO-2 (Zymed); mouse anti-AF6 (afadin; BD Biosciences); rabbit anti-Par-3 (Merck Millipore); mouse anti-MAGI-1 (ss-5), mouse anti-MAGI-3 (46) and rabbit anti-PKCζ antibodies (Santa Cruz Biotechnology); mouse anti-phospho-myosin light chain 2 (Ser19; used in Figures S2H and S3I), rabbit anti-phospho-myosin light chain 2 (Ser19; used in Figure 1C,) and rabbit anti-Ezrin (CST); rabbit anti-ROCK1 (abcam); rabbit anti-Claudin-3 (Life Technologies); mouse anti-GFP and rat anti-HA (Roche); mouse anti-DYKDDDDK (FLAG) and rat anti-E-cadherin (ECCD-2; Wako Pure Chemicals). Rat anti-activated α-catenin antibody (α18) was a kind gift from Dr. A. Nagafuchi (Nara Medical University, Nara, Japan). Rabbit anti-ASPP1 antibody and rabbit anti-ASPP2 antibody were generated by Dr. M. Hirashima.
Validation	Commercially available antibodies were validated by the manufacturers.<br>Rat anti-activated α-catenin antibody (α18) was characterized in Nagafuchi et al. (Dev. Growth Differ. 36, 59–71, 1994).<br>Rabbit anti-ASPP1 antibody and rabbit anti-ASPP2 antibody were characterized in Hirashima et al. (Dev Biol. 316, 149-59, 2008).

Eukaryotic cell lines

Policy information about cell lines

Cell line source(s)	EpH4 cells were a gift from E. Reichmann (Institute Suisse de Recherches, Lausanne, Switzerland).<br>HEK293 cells were purchased from ATCC.
Authentication	No
Mycoplasma contamination	No
Commonly misidentified lines (See ICLAC register)	No commonly misidentified cell lines were used.
